# Phonon driven charge dynamics in polycrystalline acetylsalicylic acid mapped by ultrafast x-ray diffraction

**DOI:** 10.1063/1.5079229

**Published:** 2019-01-30

**Authors:** Christoph Hauf, Antonio-Andres Hernandez Salvador, Marcel Holtz, Michael Woerner, Thomas Elsaesser

**Affiliations:** Max-Born-Institut für Nichtlineare Optik und Kurzzeitspektroskopie, 12489 Berlin, Germany

## Abstract

The coupled lattice and charge dynamics induced by phonon excitation in polycrystalline acetylsalicylic acid (aspirin) are mapped by femtosecond x-ray powder diffraction. The hybrid-mode character of the 0.9 ± 0.1 THz methyl rotation in the aspirin molecules is evident from collective charge relocations over distances of some 100 pm, much larger than the sub-picometer nuclear displacements. Oscillatory charge relocations around the methyl group generate a torque on the latter, thus coupling electronic and nuclear motions.

## I. INTRODUCTION

The interplay of electronic and nuclear motions in molecular systems is at the heart of numerous processes in physics and chemistry. In the ultrafast time domain, coherent nuclear motions have been induced by broadband vibrational and/or vibronic excitations. A vibrational wavepacket represents a nonstationary coherent superposition of quantum states in a potential determined by the electronic structure of the molecule. In the most elementary case described by the Born-Oppenheimer picture, the wavepacket undergoes a periodic oscillation in a time-independent electronic potential, i.e., nuclear and electronic motions are decoupled with a negligible impact of the nuclear motions on the shape of the vibrational potential. The coherent motion is eventually damped by vibrational decoherence induced, e.g., by the coupling of the molecule to an external bath.

A different regime of electronic and nuclear motions exists in polar and/or ionic molecular crystals with strong internal electric fields between the molecular (sub)units. The internal fields represent a major component of the total field acting locally on the individual molecular groups. As a result, the subtle nuclear rearrangements connected with vibrational excitations induce a pronounced relocation of electronic charge, in order to minimize the electrostatic energy of the crystal. Femtosecond x-ray diffraction has been applied to make such behavior directly visible.^1–3^ In the prototype material potassium dihydrogen phosphate (KH_2_PO_4_, KDP), coherent vibrational motions along a transverse-optical (TO) phonon coordinate induce a relocation of electronic charge within the PO_4_ groups and, to lesser extent, between the K^+^ ion and the PO_4_ groups.^2,3^ The length scale of charge relocation is on the order of 100 pm, i.e., a chemical bond length, while the nuclear elongations along the TO phonon coordinate are in the sub-picometer range. This hybrid character of the TO phonon is very similar to the behavior of low-frequency soft-modes in crystalline ferroelectrics which display a strong coupling to the electronic system and undergo a pronounced frequency down-shift upon the phase transition from a para- to a ferroelectric phase of the material.^4–9^

A strong coupling between electronic charge and phonon degrees of freedom makes the vibrational frequencies and absorption strength susceptible to both the local electric field strength and electronic correlation effects. Recently, this basic nonequilibrium behavior has been elucidated in nonlinear terahertz (THz) experiments with polycrystalline acetylsalicylic acid (C_9_H_8_O_4_, aspirin).^10^ Upon THz excitation of a methyl (CH_3_) rotational mode which couples strongly to the *π*-electron system of the aspirin molecules, one observes a strong blue-shift of the rotational frequency from its equilibrium value of 1.1 THz to 1.7 THz. This behavior represents a manifestation of the dynamic breakup of the strong electron-phonon correlations and has been reproduced by theoretical calculations including dynamic local-field correlations.^10,11^ While nonlinear THz spectroscopy maps the nonlinear response of the coupled system, it provides only indirect information on the electronic charge relocations connected with the vibrational excitation.

In this article, we present a study of phonon driven charge relocations in polycrystalline aspirin by femtosecond x-ray powder diffraction. Optically induced coherent motions along the methyl rotational coordinate induce strong changes in the *π*-electron system of the aspirin molecules. Electronic charge is shifted over interatomic distances on the order of 100 pm, a length scale orders of magnitude larger than the sub-picometer nuclear displacements upon CH_3_ rotation. The charge redistribution results in pronounced changes of the electronic dipole moment of the molecular units. The behavior observed here is a direct manifestation of a dynamic hybrid-mode response.

## II. EXPERIMENTAL METHODS AND RESULTS

**FIG. 1.**
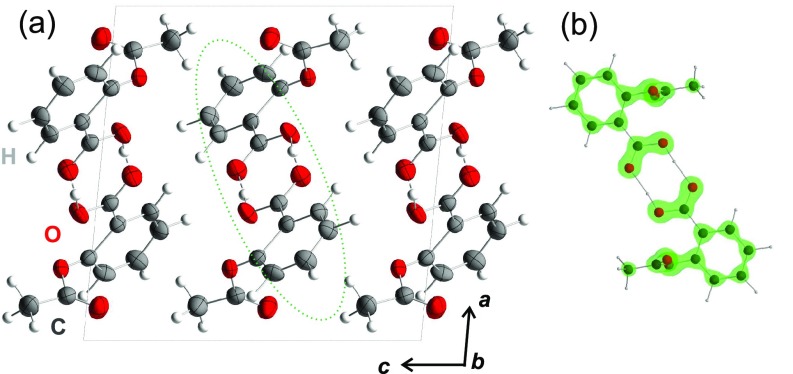
(a) Equilibrium crystal structure of aspirin viewed along the crystallographic *b*-axis, with a hydrogen bonded dimer marked with a dashed green ellipse. (b) Individual hydrogen bonded dimer with the ground state electron density displayed in an isosurface representation (green) at a value of 1800 e^−^/nm^3^.

At room temperature, aspirin crystallizes in a monoclinic crystal structure (space group P2_1_/*c*) with four formula units per unit cell [*a *=* *1.1416(5) nm, *b *=* *0.6598(2) nm, *c *=* *1.1483(5) nm, and *β* = 95.60(3)°] [Fig. 1(a)].^12–14^ In the prevailing form I of the crystallites, individual molecules form centrosymmetric hydrogen-bonded cyclic dimers as shown in Fig. 1(b), which are stacked along the crystallographic *b*-axis. The linear absorption spectra of aspirin molecules diluted in liquid solvents and of aspirin crystals display a similar pattern of electronic absorption bands, pointing to a minor electronic coupling of aspirin molecules in the crystal and a localized character of the underlying electronic excitations.^15^

The aspirin samples were prepared fresh every day from a finely ground (grain size ∼1 *μ*m) commercially available starting material (Sigma Aldrich, purity of 99.0%). Tightly pressed pellets of a thickness of ∼40 *μ*m were placed between two polycrystalline diamond windows (∼20 *μ*m thickness) and fixed on a sample holder continuously rotating around an axis parallel to the x-ray beam, with an offset of ∼300 *μ*m to mitigate potential sample damage due to the pump beam.

The ultrafast diffraction experiments are based on an optical pump/x-ray probe scheme where the sample is optically excited by 70 fs pulses with a center wavelength of 400 nm and a hard x-ray probe pulse is diffracted from the excited sample.^16^ Both pump and x-ray probe pulses are derived from an amplified Ti:sapphire laser system delivering sub-50 fs pulses centered at 800 nm with an energy of 5 mJ and a repetition rate of 1 kHz. The optical pump pulses have an energy of 25 *μ*J and are focused to a spot size of ∼500 *μ*m, providing a peak intensity I_*p*_ ∼ 2 × 10^11^ W/cm^2^ at the sample surface. The sample is electronically excited predominantly via 2-photon absorption of the pump pulses over the bandgap (*E_g_* ≈ 4.3 eV).^15^ An experimental analysis of the pump geometry of the sample shows that the fractions of pump light backscattered from and transmitted through the powder are both less than 10%. In other words, the powder layer practically absorbs all the incident pump photons. From the absorbed energy per volume, the incident pump photon flux, the molecular weight of aspirin, and the mass density of the powder sample, one estimates a fraction of 1% ± 0.5% of aspirin molecules in the irradiated sample volume which are promoted to the excited state. At this pump level, electronic excitation via nonlinear absorption processes of higher order plays a minor role. The major part (80%) of the 800 nm laser output is focused on a 20 *μ*m thin Cu tape target to generate hard x-ray pulses with a photon energy of 8.04 keV (Cu K_*α*_) and a duration of roughly 100 fs.^17^ The emitted x-ray pulses are collected, monochromatized, and focused to an ∼100 *μ*m spot size at the sample position by a Montel multilayer mirror (Incoatec) providing a flux of ∼5 × 10^6^ photons/s. Further details of this table-top femtosecond hard x-ray source and the entire experimental setup have been described earlier.^9,16,18^

**FIG. 2.**
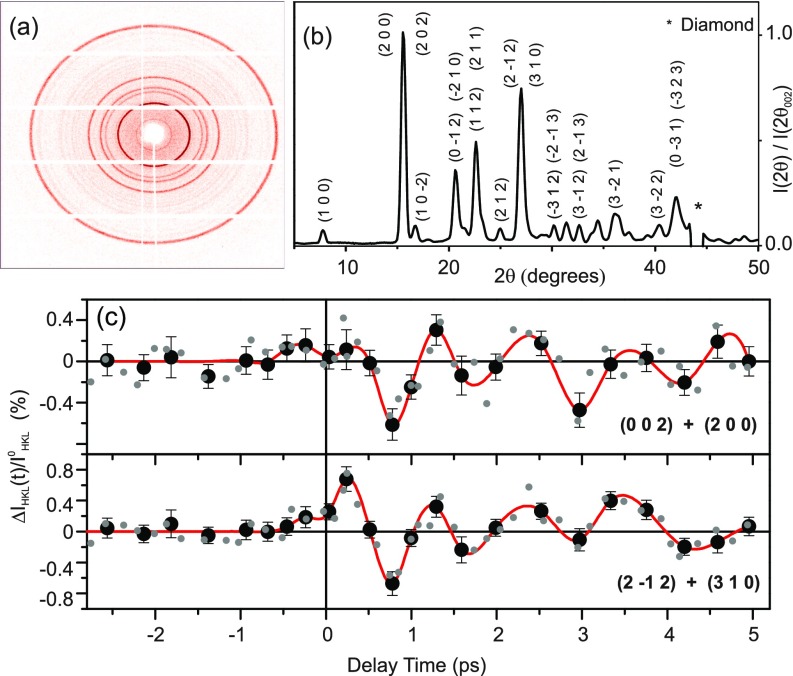
(a) Exemplary 2D powder diffraction pattern from unexcited aspirin at T = 300 K. (b) X-ray diffraction pattern obtained by integrating the 2D-pattern shown in (a) along the Debye-Scherrer rings. The normalized diffracted intensity is plotted as a function of the scattering angle 2*θ*, including salient assignments to lattice plains. (c) Change of diffracted x-ray intensity upon excitation $\Delta I_{\mathrm{hkl}}(t)/I_{\mathrm{hkl}}^{0}$ on two different Bragg reflections as a function of pump-probe delay in the femtosecond experiments at T = 300 K (black solid symbols: signal averaged over 65 individual delay positions). The grey symbols represent $\Delta I_{\mathrm{hkl}}(t)/I_{\mathrm{hkl}}^{0}$ obtained by averaging 32 neighboring delays. The solid red lines are guides to the eye.

The hard x-ray pulses probe the pump-induced structural dynamics in the photoexcited sample. The Cu K_*α*_ photons diffracted from the sample were recorded in transmission geometry by a large area detector (Pilatus Dectris 1M; pixel size 172 *μ*m × 172 *μ*m) which allows us to determine the intensity of multiple Debye-Scherrer rings at each delay time simultaneously. The powder diffraction pattern from an unexcited aspirin sample at room temperature is shown in Fig. 2(a). Integrating over all pixels with identical scattering angle 2*θ* yields 1D powder diffraction patterns as shown in Fig. 2(b), which allow for an assignment of 15 Bragg peaks to sets of lattice planes.^14^ For each individual pump-probe delay, a total integration time of the x-ray detector of 140 s was chosen. Measurements were performed in a random order over 14 days for ∼1500 different randomly generated delay times with an average 5 fs spacing in-between, covering a total delay range of 8 ps. All-optical cross correlations of the pump pulses with 800 nm pulses traveling along the optical path of the x-ray pulses were measured repeatedly to ensure a proper stacking of data from different days. These procedures in combination were chosen to mitigate the influence of potential long term fluctuations of the laser system. Finally, we sorted all individual data points according to their delay time and then averaged neighboring data points within a 250 to 400 fs interval of delay times.

Upon optical excitation, the angular positions of all observed reflections remain unchanged within the experimental accuracy and no Bragg reflections forbidden by the symmetry of the equilibrium space group P2_1_/*c* occur within our experimental sensitivity. The diffracted intensities display pronounced oscillatory changes $\Delta I_{\mathrm{hkl}}(t)/I_{\mathrm{hkl}}^{0}=(I_{\mathrm{hkl}}(t)-I_{\mathrm{hkl}}^{0})/I_{\mathrm{hkl}}^{0}$ which are plotted in Fig. 2(c) as a function of pump-probe delay [$I_{\mathrm{hkl}}(t),\;I_{\mathrm{hkl}}^{0}$: intensity diffracted with and without optical excitation] with standard errors of the mean values from averaging the raw data in the respective bins. The typical period of these oscillations is ∼1 ps, corresponding to a frequency of ∼1 THz. The binning of raw data points ensures a sufficiently high signal-to-noise ratio for a faithful reconstruction of the transient electron density via the Maximum Entropy Method detailed below. Averaging the raw data with a reduced bin size, resulting in twice the number of averaged data points, nicely retains the oscillatory signal as indicated by the light gray points in Fig. 2(c). There are no significant additional high frequency components in the transients with a reduced bin size.

## III. RECONSTRUCTION OF TRANSIENT ELECTRON DENSITY MAPS AND CHARGE DYNAMICS

The time dependent intensity changes $\Delta I_{\mathrm{hkl}}(t)/I_{\mathrm{hkl}}^{0}$ observed in the experiment are related to the transient x-ray structure factors $F_{\mathrm{hkl}}(t)$ according to $\Delta I_{\mathrm{hkl}}(t)/I_{\mathrm{hkl}}^{0}$ = (${\left|F_{\mathrm{hkl}}(t)\right|}^{2}-{\left|F_{\mathrm{hkl}}^{0}\right|}^{2})/{\left|F_{\mathrm{hkl}}^{0}\right|}^{2}$, where $F_{\mathrm{hkl}}^{0}$ are the known structure factors of the unperturbed material.^14^ The time dependent electron density ρ(r,t) averaged over all crystallites and its change relative to the unperturbed electron density $\rho_{0}(r)$ of aspirin are extracted from the structure factors $F_{\mathrm{hkl}}(t)$ by employing the maximum entropy method as implemented in the BayMEM suite of programs.^19–21^ The maximum entropy method maximizes the information entropy *S* which is defined as $S=-\sum_{\left(v=1\right)}^{N}\rho_{v}(t)\;\text{log}\;(\rho_{v}(t)/\rho_{0v})$. The summation runs over a discretized grid of *N* voxels while fulfilling a set of constraints for the supplied structure factors $F_{\mathrm{hkl}}(t)$.^19^ The treatment assumes a preservation of the initial crystal symmetry, as supported by the absence of forbidden reflections in the transient diffraction patterns. As a result, the total electronic charge on the individual aspirin entities is constant.

**FIG. 3.**
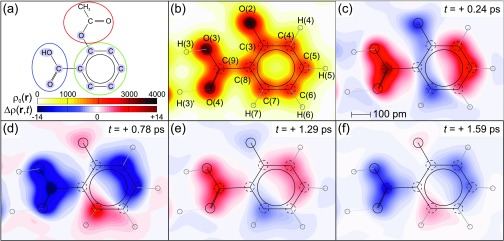
(a) Schematic drawing of an aspirin molecule, with all atoms in the plane shown in (b)–(f) highlighted in light blue. The colored circles indicate the subdivision of the molecule into the different functional groups. (b) Equilibrium electron density $\rho_{0}(r)$ and (c)–(f) transient changes in electron density Δρ(r,t) at selected delay times as two-dimensional contour maps in the plane of the C_6_ ring in steps of 100 e^−^/nm^3^ and ± 0.5 e^−^/nm^3^, respectively. The positions of the atoms in the unexcited unit cell are indicated by solid/dashed circles.

**FIG. 4.**
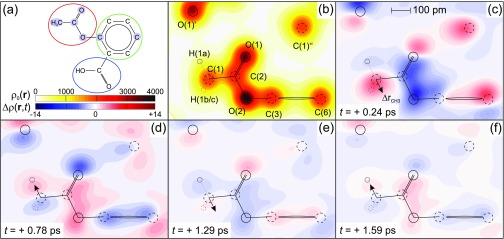
(a) Schematic drawing of an aspirin molecule, with all atoms in the plane shown in (b)–(f) highlighted in light blue. The colored circles indicate the subdivision of the molecule into the different functional groups. (b) Equilibrium electron density $\rho_{0}(r)$ and (c)–(f) transient changes in electron density Δρ(r,t) at selected delay times as two-dimensional contour maps in the plane of the acetoxy group [indicated by a red circle in (a)] in steps of 100 e^−^/nm^3^ and ± 0.5 e^−^/nm^3^, respectively. The positions of the atoms in the unexcited unit cell are indicated by solid/dashed circles. Primes (′) denote atoms belonging to other molecules. In the CH_3_ group, only the hydrogen atom H(1a) is located within the displayed plane. The projected position of the two other hydrogen atoms is denoted with a pointed circle. The direction of the momentary displacement of the methyl group $\Delta d_{\text{CH}3}$ is schematically indicated by an arrow.

In Fig. 3, equilibrium and transient charge density maps are summarized for the plane containing the C_6_ rings and the COOH carboxy groups of the aspirin molecules, highlighted by green and blue circles in Fig. 3(a). Figure 4 displays analogous maps in the plane of the CH_3_CO_2_ acetoxy group, highlighted by a red circle in Fig. 4(a). Figures 3(b) and 4(b) show the equilibrium charge density $\rho_{0}(r)$, while Figs. 3(c)–3(f) and 4(c)–4(f) display differential charge densities $\Delta \rho (r,t)=\rho (r,t)-\rho_{0}(r)$ for different pump-probe delays. The absolute values of Δρ(r,t) have an uncertainty of up to 1.7 e^−^/nm^3^. The differential charge densities reveal a pronounced modulation of charge density with time, close to the original positions of the lattice atoms, which are indicated by black circles. It is important to note that all major changes Δρ(r,t) are centered on the ground state atomic positions, without a charge transfer to previously unoccupied positions in space. This behavior demonstrates that the molecular arrangement of the ground state crystal structure is preserved and chemical processes are absent, in contrast to other polar materials such as paraelectric ammonium sulfate (NH_4_)_2_SO_4_.^22^

**FIG. 5.**
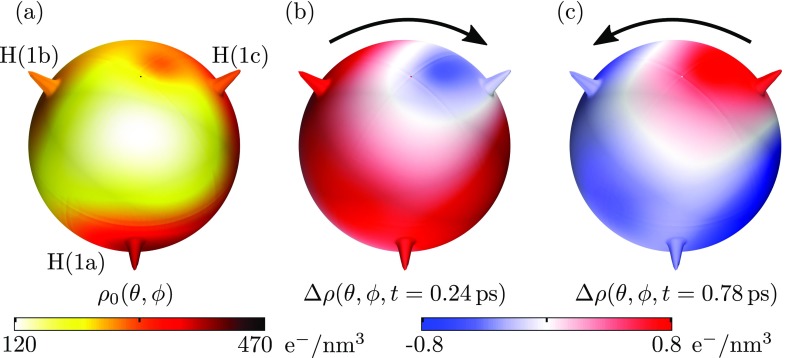
Integrated charge densities in e^−^/nm^3^ in a spherical shell with a radius of 150 pm and a thickness of 50 pm around the central C atom of the methyl group in dependency of the polar angles *θ* and *ϕ* (a) for the ground state density distribution and (b) and (c) two differential charge density distributions at selected delay times. The value of the (differential) charge density is given by the color coding. In all three cases, the viewing direction is chosen along the C-C bond and filet indentations are shown at the solid angles corresponding to the proton positions. The arrows schematically indicate the direction of the net torque on the methyl group.

In order to gain insight into the coupling between the electronic charge density oscillations and the rotation of the methyl group, we investigated the charge dynamics in a spherical shell around the methyl group in more detail. To this end, we integrated both the stationary and the differential charge density in a spherical shell which is centered at the carbon atom, with a radius of 150 pm and a gaussian radial profile of 50-pm thickness. Figure 5 shows the results plotted in a spherical coordinate system characterized by the angles *θ* and *ϕ*. The spheres display filet indentations at the solid angles corresponding to the proton positions. The color code represents the stationary charge density [panel (a)] and differential charge density maps [panels (b) *t* = +0.24 ps and (c) *t* = +0.78 ps] as a function of *θ* and *ϕ*. Both the stationary electron density and its changes as a function of the pump-probe delay are distinctly asymmetric with respect to the proton positions. In particular, the transient excess charge opposing the protons on the shell is concentrated close to one of the protons with a maximum at a distinctly different solid angle. As a result, the electronic charge density oscillation exerts a net torque on the methyl group which is the basis for the strong coupling between the electronic charge density oscillations and the rotation of the methyl group in the phonon mode of aspirin.

The Δρ(r,t) maps in Figs. 3 and 4 reveal an oscillatory relocation of charge density involving the electron system of the entire molecule, which is particularly pronounced within the plane containing the carboxy group and the benzene ring. To quantify the dynamics of this oscillatory electron relocation in more detail, we determined the change of the integrated charge Δ*Q*(*t*) on individual atoms and different functional groups. To derive Δ*Q*(*t*) on different structural units, the entire unit cell was sub-divided into sub-volumes containing one atom each. This partitioning is based on the unperturbed crystal structure and each point in the unit cell is assigned to the atom nearest to it. Transient atomic charges were then obtained by integration over the respective subvolumes. The charge of the hydrogen atoms was added to the charge of the heavy atoms they are bound to, since their contributions cannot be properly distinguished at the resolution provided by the experiment.

**FIG. 6.**
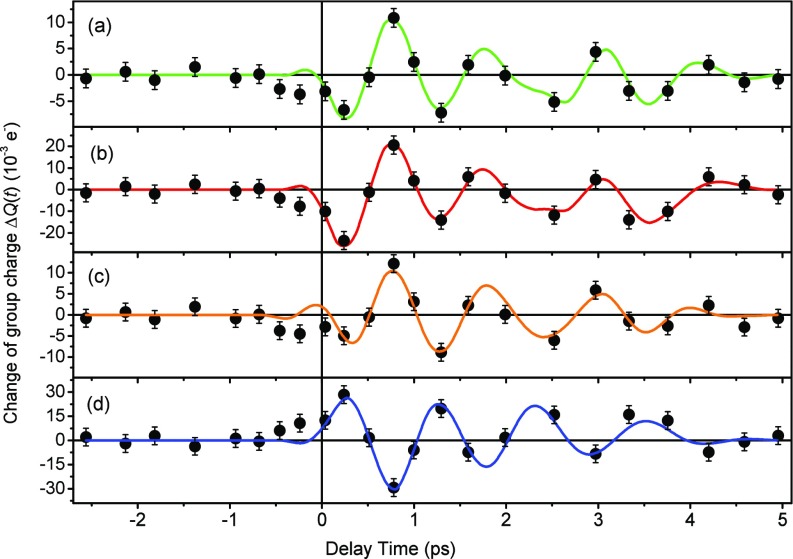
Spatially integrated group charge changes ΔQ(t) of (a) the benzene ring, (b) the entire CH_3_CO_2_ acetoxy group, (c) solely the CH_3_ methyl group, and (d) the carboxy group plotted as a function of pump-probe delay (black symbols). Colored solid lines: At the individual data points, the solid line has exactly the value of the corresponding piecewise fit. Between two data points, the solid lines are linear interpolations of the two overlapping piecewise fits of neighboring sets of data points.

The results presented in Fig. 6 reveal pronounced oscillatory changes of local charge on the benzene ring, the acetoxy group, and the carboxy group as a function of delay time. The oscillation frequency is derived from a piece-wise fit of a sinusoidal function to the data points in the time-resolved transients (solid lines). This procedure results in a total of 9 momentary frequencies for each data point with a positive delay time which is the midpoint of an interval of seven neighboring delay times. The average oscillation frequency has a value of 0.9 ± 0.1 THz. The stated uncertainty describes the range of frequency values occurring during the decay of the oscillation. The charge changes on the benzene ring and the acetoxy group occur in phase, while the charge on the carboxy group changes with the opposite phase. This behavior is equivalent to an oscillatory intramolecular charge relocation between the carboxy group and the rest of the molecules and connected with changes of the electronic dipole moment along the axis connecting the carboxy group and the benzene ring. The amplitude of the charge oscillations averaged over all aspirin molecules is on the order of 1% of an elementary charge. Assuming charge transfer only on the excited molecules and taking into account the 1% fraction of excited molecules, the oscillation amplitude per excited molecule is on the order of one elementary charge *e*, corresponding to a transient change of the electric dipole moment on the order of 1 Debye along the axis connecting the ring structure and the carboxy group. The oscillations are severely damped on a time scale of several picoseconds. This phenomenon is mainly caused by inhomogeneous broadening of phonon frequencies in the polycrystalline sample, as has been discussed in the context of the nonlinear THz experiments on aspirin.^10,23^

## IV. DISCUSSION

The time resolved x-ray diffraction data and the transient charge density maps derived from them reveal periodic motions of electronic charge with an oscillation frequency of 0.9 ± 0.1 THz. The oscillations are due to coherent nuclear motions which are generated upon electronic excitation of the aspirin crystallites by the pump pulse. The absence of a time delay in the onset of the oscillations points to an impulsive displacive driving mechanism.^22,24^ In this process, the change of the molecular electron distribution in the excited compared to the ground state modifies the potential of vibrational and rotational modes and induces coherent motions via the electronic deformation potential.

The observed oscillation frequency of 0.9 ± 0.1 THz is very close to the methyl rotation frequency in the electronic ground state. In addition to the methyl rotation, there are a number of other low-frequency modes, among them the intermolecular hydrogen bond modes of the aspirin dimer structure (Fig. 1)^10,25,26^ which, however, occur at higher frequencies. The frequency of the methyl rotation is strongly red-shifted compared to that of a free methyl rotator, a consequence of its strong coupling to the electronic system.^10^ We conclude that the charge oscillations are due to coherent methyl rotations in the crystallites.

The transient electron density maps of Figs. 3 and 4 demonstrate charge relocations on a length scale of 100 pm, comparable to the interatomic distances in the molecular structure. Such distances are much larger than the sub-picometer displacements of vibrational and/or rotational elongations. The mismatch of length scales represents a hallmark of the impact of a hybrid mode on the electronic system.^2,9^ The strong correlation of nuclear and electronic degrees of freedom and the presence of strong local field effects make the polarizable charge distribution highly sensitive to minute changes in atomic positions. The observed behavior bears strong similarity to the early qualitative picture of soft-modes developed by Cochran,^4,5^ here with quantitative insight into charge distributions and their dynamics.

**FIG. 7.**
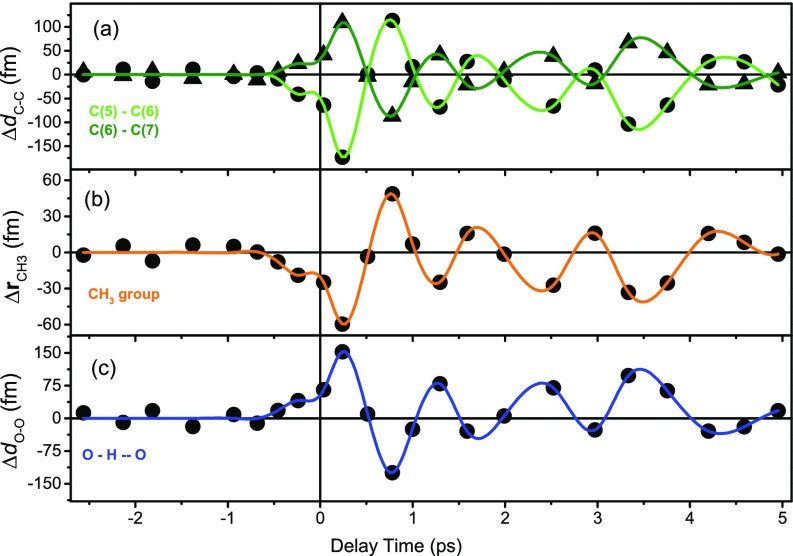
(a) Time dependent changes of selected interatomic distances $\Delta d_{C-C}$ in the benzene ring, (b) transient changes of the position of the methyl group Δ**r**_CH3_, and (c) the intermolecular oxygen-oxygen distance $\Delta d_{O-O}$ along an O-H ··· O hydrogen bond of the aspirin dimer, plotted as a function of pump-probe delay (black symbols). The colored lines are guides to the eye only.

To further validate this picture, we retrieved the transient positions of the carbon and oxygen atoms in the aspirin entities from the time dependent electron density ρ(r,t), in particular from the core electron density which follows the motion of the respective nucleus. This is done by fitting a three-dimensional gaussian distribution to the high core electron density in the transient electron density maps. The sub-picometer displacements connected with the 0.9 ± 0.1 THz methyl rotations are complemented by motions of the carbon and oxygen atoms in the aspirin molecule with similar amplitudes (Fig. 7). As is evident from the data in Fig. 4, the hydrogen atoms of the CH_3_ groups are not discernible with the resolution provided by the experimental density maps and, thus, one cannot follow their motion upon excitation. Instead, the transient in Fig. 7(b) reflects the motion of the entire CH_3_ group from its ground state position Δ**r**_CH3_. An analysis of its momentary position reveals that it mainly oscillates along a line roughly perpendicular to the C(1)-C(2) bond and roughly parallel to the C(1)-H(1a) bond in the plane of Fig. 4 (schematically indicated by an arrow).

The onset of the carbon displacements in Fig. 7(a) and of the methyl motion shown in Fig. 7(a) occurs in phase within the first 0.25 ps, i.e., there is no mutual delay in the nuclear response of different parts of the aspirin molecules. This observation is in line with a picture in which the relocation of electronic charge follows the methyl displacement adiabatically, leading to a synchronous change of the vibrational potential along different atomic coordinates. As the sub-50 fs time scale of carbon motions is much shorter than the methyl rotation period, the different nuclei adopt to their momentary position well within the time resolution of the present experiment and the motions appear to be in phase. It should be noted that the maximum change of the total charge on the methyl group occurs only in the second half cycle of the methyl oscillation [Fig. 6(c)], suggesting that the contribution of the methyl group to the total periodically shifted charge is limited.

A transient position change of the carboxy group affects the hydrogen bond length in the aspirin dimer. To assess this potential geometry change, we plot the transient change of the OH⋯O distance $\Delta d_{O-O}$ in Fig. 7(c). The absolute displacements are similar to those of the other atoms and of the methyl group, i.e., the elongation by several hundreds of femtometers is small compared to the length of the hydrogen bonds of 262 pm. Thus, there are minor changes of hydrogen bond strength upon excitation of the methyl rotation.

In conclusion, the hybrid-mode character of the 1-THz methyl rotation in polycrystalline aspirin is manifested in pronounced relocations of electronic charge on a length scale of interatomic distances or covalent chemical bonds. The present study adds in-depth structural information to recent work in which the nonlinear vibrational response in the electronic ground state of aspirin has been studied by ultrafast two-dimensional THz spectroscopy in conjunction with high level theoretical calculations.^10^ A combination of THz excitation with x-ray diffraction probing holds strong potential to unravel the hybrid-mode-induced charge dynamics in the ground state of aspirin and soft-mode dynamics in ferroelectric materials.

## SUPPLEMENTARY MATERIAL

See supplementary material for an animation that illustrates the phonon driven oscillatory charge relocation in an acetylsalicylic acid molecule.

## References

[1] T. Elsaesser and M. Woerner , “ Perspective: Structural dynamics in condensed matter mapped by femtosecond x-ray diffraction,” J. Chem. Phys. 140, 020901 (2014). 10.1063/1.4855115 24437858

[2] F. Zamponi , P. Rothhardt , J. Stingl , M. Woerner , and T. Elsaesser , “ Ultrafast large-amplitude relocation of electronic charge in ionic crystals,” Proc. Nat. Acad. Sci. U. S. A. 109, 5207–5212 (2012). 10.1073/pnas.1108206109 PMC3325693 22431621

[3] F. Zamponi , J. Stingl , M. Woerner , and T. Elsaesser , “ Ultrafast soft-mode driven charge relocation in an ionic crystal,” Phys. Chem. Chem. Phys. 14, 6156 (2012). 10.1039/c2cp24072f 22441549

[4] W. Cochran , “ Crystal stability and the theory of ferroelectricity,” Phys. Rev. Lett. 3, 412–414 (1959). 10.1103/PhysRevLett.3.412

[5] W. Cochran , “ Crystal stability and the theory of ferroelectricity,” Adv. Phys. 9, 387–423 (1960). 10.1080/00018736000101229

[6] T. P. Dougherty , G. P. Wiederrecht , K. A. Nelson , M. H. Garrett , H. P. Jenssen , and C. Warde , “ Femtosecond time-resolved spectroscopy of soft modes in structural phase transitions of perovskites,” Phys. Rev. B 50, 8996–9019 (1994). 10.1103/PhysRevB.50.8996 9974941

[7] S. Fahy and R. Merlin , “ Reversal of ferroelectric domains by ultrashort optical pulses,” Phys. Rev. Lett. 73, 1122–1125 (1994). 10.1103/PhysRevLett.73.1122 10057630

[8] M. Kozina , T. van Driel , M. Chollet , T. Sato , J. M. Glownia , S. Wandel , M. Radovic , U. Staub , and M. C. Hoffmann , “ Ultrafast X-ray diffraction probe of terahertz field-driven soft mode dynamics in SrTiO_3_,” Struct. Dyn. 4, 054301 (2017). 10.1063/1.4983153 28503632 PMC5415405

[9] C. Hauf , A. Hernandez Salvador , M. Holtz , M. Woerner , and T. Elsaesser , “ Soft-mode driven polarity reversal in ferroelectrics mapped by ultrafast x-ray diffraction,” Struct. Dyn. 5, 024501 (2018). 10.1063/1.5026494 29657958 PMC5889304

[10] G. Folpini , K. Reimann , M. Woerner , T. Elsaesser , J. Hoja , and A. Tkatchenko , “ Strong local-field enhancement of the nonlinear soft-mode response in a nonlinear crystal,” Phys. Rev. Lett. 119, 097404 (2017). 10.1103/PhysRevLett.119.097404 28949583

[11] A. M. Reilly and A. Tkatchenko , “ Role of dispersion interactions in the polymorphism and entropic stabilization of the aspirin crystal,” Phys. Rev. Lett. 113, 055701 (2014). 10.1103/PhysRevLett.113.055701 25126928

[12] P. J. Wheatley , “ Crystal and molecular structure of aspirin,” J. Chem. Soc. 0, 6036–6048 (1964). 10.1039/jr9640006036

[13] A. D. Bond , R. Boese , and G. R. Desiraju , “ On the polymorphism of aspirin: Crystalline aspirin as intergrowths of two ‘polymorphic’ domains,” Angew. Chem. Int. Ed. 46, 618–622 (2007). 10.1002/anie.200603373 17139692

[14] C. Wilson , “ Interesting proton behaviour in molecular structures. Variable temperature neutron diffraction and ab initio study of acetylsalicylic acid: Characterising librational motions and comparing protons in different hydrogen bonding potentials,” New J. Chem. 26, 1733–1739 (2002). 10.1039/b203775k

[15] M. Vala, Jr. and J. Tanaka , “ The polarized crystal absorption spectrum of aspirin,” J. Mol. Spectrosc. 32, 169–180 (1969). 10.1016/0022-2852(69)90212-4

[16] F. Zamponi , Z. Ansari , M. Woerner , and T. Elsaesser , “ Femtosecond powder diffraction with a laser-driven hard x-ray source,” Opt. Express 18, 947–961 (2010). 10.1364/OE.18.000947 20173917

[17] F. Zamponi , Z. Ansari , C. V. Korff Schmising , P. Rothhardt , N. Zhavoronkov , M. Woerner , T. Elsaesser , M. Bargheer , T. Trobitzsch-Ryll , and M. Haschke , “ Femtosecond hard X-ray plasma sources with a kilohertz repetition rate,” Appl. Phys. A 96, 51–58 (2009). 10.1007/s00339-009-5171-9

[18] M. Holtz , C. Hauf , J. Weisshaupt , A. Hernandez Salvador , M. Woerner , and T. Elsaesser , “ Towards shot-noise limited diffraction experiments with table-top femtosecond hard x-ray sources,” Struct. Dyn. 4, 054304 (2017). 10.1063/1.4991355 28795079 PMC5517321

[19] S. Smaalen , L. Palatinus , and M. Schneider , “ The maximum-entropy method in superspace,” Acta Cryst. A 59, 459–469 (2003). 10.1107/S010876730301434X 12944610

[20] M. Woerner , M. Holtz , V. Juvé , T. Elsaesser , and A. Borgschulte , “ Femtosecond x-ray diffraction maps field-driven charge dynamics in ionic crystals,” Faraday Disc. 171, 373–392 (2014). 10.1039/C4FD00026A 25415431

[c21] 21.Following an established procedure described in detail in Ref. 20, we started from the known unperturbed electron density $\rho_{0}(r)$ of aspirin as derived from a high quality steady state determined by a single crystal neutron diffraction experiment described in Ref. 14 at room temperature and accounted for the limited angular resolution of the ultrafast diffraction experiment.

[22] M. Woerner , F. Zamponi , Z. Ansari , J. Dreyer , B. Freyer , M. Prémont-Schwarz , and T. Elsaesser , “ Concerted electron and proton transfer in ionic crystals mapped by femtosecond x-ray powder diffraction,” J. Chem. Phys. 133, 064509 (2010). 10.1063/1.3469779 20707577

[23] R. Ruppin , “ Surface effects on optical phonons and on phonon-plasmon modes,” Surf. Sci. 34, 20–32 (1973). 10.1016/0039-6028(73)90184-2

[24] M. Bargheer , N. Zhavoronkov , Y. Gritsai , J. C. Woo , D. S. Kim , M. Woerner , and T. Elsaesser , “ Coherent atomic motions in a nanostructure studied by femtosecond X-ray diffraction,” Science 306, 1771–1773 (2004). 10.1126/science.1104739 15576618

[25] M. Boczar , M. J. Wójcik , K. Szczeponek , D. Jamróz , A. Zieba , and B. Kawalek , “ Theoretical modeling of infrared spectra of aspirin and its deuterated derivative,” Chem. Phys. 286, 63–79 (2003). 10.1016/S0301-0104(02)00912-6

[26] N. Laman , S. S. Harsha , and D. Grischkowsky , “ Narrow-line waveguide terahertz time-domain spectroscopy of aspirin and aspirin precursors,” Appl. Spectrosc. 62, 319–326 (2008). 10.1366/000370208783759768 18339241

